# Pilocarpine in Bright’s Disease

**Published:** 1888-04

**Authors:** 


Pilocarpine in Bright's Disease.—The Vienna correspondent of the *British Medical Journal* says that Drs. Benezür and C. Csatáry of Buda-Pesth, give in a recent number of *Orvosi Hetilap* the following summary of a series of articles on the effect of pilocarpine chloride in Bright's disease:

1. The patients become accustomed to the pilocarpine, and even large doses, such as six centig., do not at a later period produce such disagreeable after-effects as doses of one centig. at the beginning of the treatment. The injections of pilocarpine should not be discontinued in consequence of symptoms that had been considered as being dangerous. 2. The effect of pilocarpine on the daily secretion of saliva, sweat, and urine, as well as on the daily oscillations of the amount of hæmoglobin in the blood, is in most cases regulated by the stage of the disease and the quantity of liquid that had been taken. 3. The œdema disappeared the more rapidly the larger the dose of pilocarpine given, and the less the quantity of liquid that the patient had taken. 4. Pilocarpine considerably increases the density of the blood for from four to five hours. 5. The hydræmia in Bright's disease does not depend on the amount of œdema. 6. The quantity of hæmoglobin in the blood diminishes; that is to say, the hydræmia increases when the general condition of the patient becomes impaired during the progress of the disease. 7. When used according to the above-mentioned principles, pilocarpine will be found in most cases of Bright's disease, even when hot baths and diaphoretics prove useless, always to diminish dropsy to such an extent that the patient is more or less protected against dangerous uraæmic suffocative attacks. In this way it may be possible to effect a relative cure; that is, in secondary granular contracted kidney.

